# From the Laboratory to The Vineyard—Evolution of The Measurement of Grape Composition using NIR Spectroscopy towards High-Throughput Analysis

**DOI:** 10.3390/ht8040021

**Published:** 2019-11-30

**Authors:** Aoife Power, Vi Khanh Truong, James Chapman, Daniel Cozzolino

**Affiliations:** 1 Technological University Dublin, Dublin, Ireland; power.aoife@gmail.com; 2 Nanobiotechnology Laboratory, School of Science, College of Science, Engineering and Health, RMIT University, Melbourne VIC 3001, Australia; vi.khanh.truong@rmit.edu.au (V.K.T.); james.chapman@rmit.edu.au (J.C.); 3 Food Science and Technology, Bundoora Campus, School of Science, College of Science, Engineering and Health, RMIT University, Melbourne VIC 3086, Australia

**Keywords:** infrared, grapes, composition, viticulture, chemometrics

## Abstract

Compared to traditional laboratory methods, spectroscopic techniques (e.g., near infrared, hyperspectral imaging) provide analysts with an innovative and improved understanding of complex issues by determining several chemical compounds and metabolites at once, allowing for the collection of the sample “fingerprint”. These techniques have the potential to deliver high-throughput options for the analysis of the chemical composition of grapes in the laboratory, the vineyard and before or during harvest, to provide better insights of the chemistry, nutrition and physiology of grapes. Faster computers, the development of software and portable easy to use spectrophotometers and data analytical methods allow for the development of innovative applications of these techniques for the analyses of grape composition.

## 1. Introduction

Rapid analytical methods have the potential to deliver high-throughput options in both research and real-life applications. Vibrational spectroscopy techniques (near infrared (NIR), mid infrared (MIR)) are well known to successfully represent the “fingerprint” of the sample analysed [1,2,3,4,5,6]. These techniques could be implemented to simplify analytical methods and decrease cost and time of analysis [1,2,3,4,5,6]. State of the art technology, instrumentation (e.g., hand-held, portable), software and data mining methods are the key drivers for the increased adaptation/utilisation of these methods in research and development (R&D) for high-throughput applications in crops and in the vineyard. 

**Figure 1:**
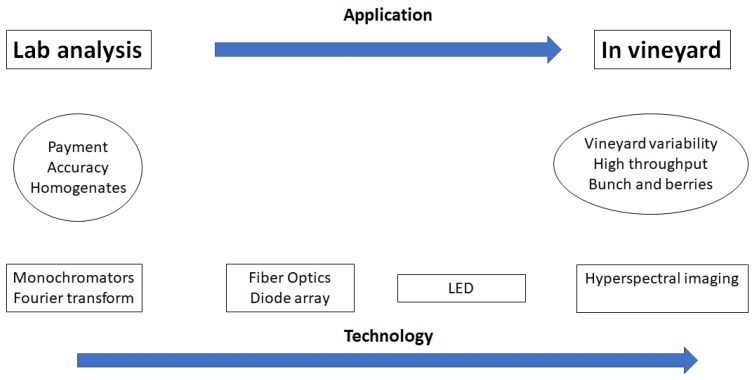
Evolution on the measurement of grape composition from the laboratory to the vineyard towards high-throughput analysis.

The use of visible (VIS) and NIR spectroscopy has been evaluated as a laboratory method to measure grape compositional parameters extensively in the literature [1,2,3,4,5,6]. The main objective of the laboratory applications was on the accurate determination of sugars and colour (e.g., total anthocyanins) in grapes, mainly for commercial purposes [1,2]. This approach has been implemented by the wine industry through the collection of the spectra of homogenised grape samples with commercial laboratory spectrophotometers [2,3,4,5,6]. Nowadays, the measurement of grape composition has moved from the laboratory to the vineyard where researchers have demonstrated alternative sample presentation modes combined with adaptable and portable instruments that allowed for a more cost-effective analysis of grapes at the vineyard and the weighbridge (Figure 1). In the last decade an increased interest in NIR applications has been boosted by numerous factors including the availability of handheld, portable and miniaturised instruments and hyperspectral (HYPER) cameras and developments in data analytical methods [2,3,4,5,6]. More recently, the use of HYPER systems has also allowed for the spatiotemporal collection of data with more opportunities to analyse different aspects of grape and plant composition, nutrition, and physiology in the vine and vineyard.

The last 20 years have seen an increase in innovative applications of these techniques for the analyses of grape composition moving from the laboratory to the vineyard. This review summarises and discusses the potential of NIR spectroscopy coupled with HYPER imaging for the analysis of grape composition (e.g., single grapes, whole or intact bunch) in the vineyard as a high-throughput method.

## 2. In Vineyard Applications (Single Berries, Whole Bunch)

The chemical composition of grapes varies across the vineyard as result of soil variability (e.g., nutrients and water), fertilization, climate and other environmental conditions. This variability creates opportunities to either exploit the inherent variations to produce different wines with different qualities or to apply precision viticultural methods to minimise the variability to optimise inputs and ultimately the output (improve wine quality) [2,3,4,5,6]. The combination of VIS and NIR spectroscopy has been widely reported as a laboratory technique to evaluate grape compositional parameters [1,2,3,4,5,6].

Schaare and collaborators [2] have reported the utilisation of VIS-NIR spectroscopy to evaluate the chemical composition of grapes during machine harvesting and on the use of this information to generate spatial distribution maps associated with quality to improve viticultural practices [2]. The authors described the use of a commercial VIS and NIR instrument to quantify the total soluble solids (TSS) in *Sauvignon Blanc* grapes in motion on a conveyor belt. The TSS content of the individual berries was predicted with the author’s models yielding a coefficient of determination (R^2^) of 0.83 and a root mean square standard error of prediction (RMSEP) of 1.10 °Brix, respectively [2]. The same authors also developed a partial least squares (PLS) discriminant (DA) regression to differentiate the composition of the grapes based on the VIS-NIR spectra of the grapes as they progressed on the conveyor belt [2].

Gonzalez–Caballero and co-workers investigated the capability of NIR spectroscopy to monitor grape ripeness associated with bunch orientation and position, with the objective to optimise harvesting [7,8]. These authors used a handheld instrument with a range of 1600 to 2400 nm, to distinguish clusters of cv. *Pedro Ximénez* and *Cabernet Sauvignon* [7,8]. The authors observed that the NIR spectra of the grape samples was dependent on the position and orientation of each individual bunch [7,8]. Therefore, NIR spectroscopy enabled analysts to distinguish between the fruits ripening stages, early, middle, and late in addition to categorising the grapes based on their TSS content using PLS-DA regression (correct classification rates ranged between 79% and 88%). The authors concluded that NIR spectroscopy in the 1600–2400 nm range was useful for the vineyard-based monitoring of the grape development process, which could allow for the selective harvesting of fruit to produce different styles of wine [7,8]. In an earlier study, the same researchers reported the application of NIR spectroscopy to monitor variations in the internal compositional properties of *Vitis vinifera* L. during development and at maturity [7,8]. The authors reported that NIR combined with two regression techniques both modified PLS (MPLS) and LOCAL regression allowed for the analysis of grapes in clusters. The information generated by the author’s models was demonstrated to quantify changes in the TSS, pH, tartaric acid (TA), malic acid (MA) and potassium (K) content of the fruit, with comparable results to the more traditional means of monitoring [7,8].

Recently, Wample and collaborators described the combination of global positioning and information systems (GPS and GIS) with NIR spectroscopy to quantify both the anthocyanins (Antho) and TSS content in wine grapes at two separate vineyards in California [9]. The researchers demonstrated that the application of such technologies could allow wineries to produce two wines of different quality, rather than a single blended wine, which improved the overall income of the winery [9].

Fourier transform (FT) NIR spectroscopy was evaluated by Aleixandre-Tudo and co-authors for the “on-line” analysis of grapes either when the fruits were being transferred via conveyor belt or at a “static” sampling system [10]. The authors reported RMSEP and residual predictive deviation (RPD) values of 12% and 2.37, 12.3% and 3.37, 7.8% and 3.2, 16.7% and 2.84 for tannins (TAN) (mg g^−1^), Antho (mg g^−1^) on a fresh weight basis, total phenols (TPhenol), and colour density (AU), respectively [10]. Moreover, the authors findings demonstrated the ability of NIR spectroscopy to monitor TPhenol composition of grape samples on a conveyor belt, which could ultimately allow the development of an automated online analytical system [10].

Ferrer–Gallego and collaborators described the application of NIR spectroscopy to quantify TPhenol compounds in *Vitis vinifera* L. cv. *Graciano* [11,12]. The authors measured TPhenol content in whole berries and grape skins at different stages of maturation [11]. The studies calibration models demonstrated their effectiveness in measuring the fruits flavonols (variation between the standard HPLC method and the NIR approach was 7.8% and 10.7%) in whole grapes and skins. The authors highlighted that the best models were found by collecting the spectra of the intact grapes using a fibre-optic probe, which the authors attributed to the probe versatility, which removed the necessity for sample manipulation prior to individual readings [11,12]. This study also highlighted the potential of the process to provide a rapid cost-effective method of analysis [11]. In a similar study, the same authors reported that NIR spectroscopy possess good potential as a means of predicting multiple sensory parameters (sourness, astringency, tannic intensity, dryness, and hardness), colour and aroma intensity in both grape seed and skin samples [11,12]. These researchers demonstrated how the NIR spectroscopy analysis results were comparable to those determined by a conventional sensory panel of winemakers. The reported externally validated difference between the classical sensory panel and the NIR method’s findings being 4.5% for hardness and 8.7% for colour in the case of seeds and for skins 9.8% for tannic intensity and 13.7% for astringency [11,12].

Barnaba and colleagues evaluated the use of an acousto-optic tunable filter near infrared (AOTF–NIR) instrument to monitor the ripening of Sangiovese grapes sourced from four separate Italian vineyards over three consecutive seasons [13]. The authors used PLS regression to predict several parameters such as TSS (°Brix), total sugars (g L^−1^), glucose (g L^−1^), fructose (g L^−1^), density (g mL^−1^), TA (g L^−1^), tartaric acid (g L^−1^), pH, MA (g L^−1^), gluconic acid (g L^−1^), yeast assimilable nitrogen (mg L^−1^), Antho (mg L^−1^) and TPhenol (mg L^−1^) [13]. Barnaba and co-authors concluded that AOTF–NIR spectroscopy was useful for the direct infield determination of both Sangiovese grape quality and the fruits ripening profile, which would be of benefit to the viticulture industry. Torchio and collaborators also investigated the feasibility of predicting the extractable phenolic content of intact grape seed samples using FT-NIR spectroscopy [14]. Their calibration models were shown to be capable of quantitative analysis of the samples’ total flavonoids, pro-anthocyanidins, galloylation percentage as well as their low molecular weight flavanols, such as catechin, epicatechin and procyanidin [14].

Muganu and collaborators employed a non-destructive AOTF-NIR instrument to investigate the impact of soil management practices (e.g., tillage, natural vegetation) on vine growth and composition of *Canaiolo nero* and *Trebbiano giallo* grapes over two consecutive vintage sessions (2010–11) [15]. The NIR data was used to predict TSS, pH, TA, Antho and TPhenol content of the grape samples [15]. The researchers emphasised that AOTF-NIR spectroscopy coupled with multivariate data analysis demonstrated great potential as a means of rapidly assessing grape quality, which, along with vine characteristics, was influenced by soil management practices [15].

Wenzhong and collaborators developed a method to monitor the deterioration of grapes using FT-MIR spectroscopy together with chemometrics by predicting volatiles compounds directly in the whole grapes measured by spectroscopy (e.g., deteriorated *vs.* fresh) [16]. The authors established that the rate at which volatiles were released from the grapes was greatest for samples that had just started to deteriorate based on their IR spectra [16]. The authors proposed that the study could allow for the development of a rapid, non-destructive, low cost sensor to establish the quality of fruits in large storage facilities. Boido and collaborators investigated the ability of NIR spectroscopy and chemometrics to quantify several glycosylated aroma compounds in *Tannat* grape samples [17]. The authors demonstrated that NIR spectroscopy may be applied as screening tool for the rapid analysis of the fruits glycosylated compounds and would contribute to improve management decisions at the vineyard and the winery [17]. Yang and collaborators also reported the combination of UV-VIS-NIR spectroscopy with PLS-DA as a promising method for the non-destructive classification of grape seed varieties (210–1100 nm) [18].

Rolle and co-workers used FT-NIR spectra and texture parameters to quantify both the TPhenol content and extractability (EXPHENOL) of intact *Cabernet Sauvignon* grape seeds [19]. The TPhenol content was predicted with a standard error prediction (SEP) of less than 8%, using FT-NIR spectroscopy [19]. The study inferred that the rapid analytical methods could be utilised during winemaking to rapidly monitor the seeds phenolic maturity [19]. A study by Lv and collaborators reported the ability of VIS and NIR spectroscopy to categorise grape ripeness [20]. These experiments proved the ability of NIR spectroscopy combined with different chemometric method to monitor grape maturity [20]. Validation of the results showed that the combination of linear discriminant analysis (LDA) with principal component analysis PCA and PLS achieved classification accuracy of 100% [20]. The researchers concluded that VIS and NIR spectroscopy has significant potential as a rapid identification technique to monitor the natural variation in grape composition through ripeness and at harvest [20]. 

Urraca and collaborators demonstrated the viability of the combination of a portable FT-NIR instruments and chemometrics to successfully determine the TSS of grape berries through the comparison of laboratory and in field measurements [21]. The RMSEP = 1.68 °Brix, and SEP = 1.67 °Brix developed with the NIR spectra collected in the vineyard were sufficiently like those obtained using a laboratory instrument (RMSEP = 1.42 °Brix, SEP = 1.40 °Brix) [21]. The authors concluded that a protocol can be defined for in vineyard assessment of TSS grapes by NIR spectral analyser [21]. Xiao and co-workers also compared the findings of a benchtop FT-NIR and portable grating scanning instrument measuring TSS content in *Ruby* seedless grape berries [22]. These authors determined that a modified piecewise direct standardization (PDS) transfer method could be generated to transfer the calibration between the benchtop FT-NIR spectrometer and the portable grating scanning spectrometer, with this linear interpolation-PDS removing the difficulty caused by the instruments resolution difference, and would ultimately allow portable scanning instrument to perform better than a more traditional wavelengths-reserved method [22].

Heredia and co-workers used a portable NIR instrument (908–1676 nm) to access the capability of similar portable instruments to determine the levels of extractable phenolic compounds of red grapes (*Vitis vinifera* L.), through the collection of the spectra of whole grapes and skins at harvest in two vintages (2016 and 2017) [23]. However, the authors highlighted that some issues can influence the ability of the NIR instrument to measure grape composition in the “in vineyard” for EXPHENOL [23]. These authors emphasised the importance of considering environmental, plant physiological, and other conditions that can hamper the robustness of the models used to predict EXPHENOL content [23].

Costa and co-authors reported the use of VIS-NIR reflectance spectroscopy as an effective tool for the non-destructive assessment of grape quality. The authors collected the spectra of the berries of both *Shiraz* and *Cabernet Sauvignon* grape varieties using a portable instrument (450 to 1800 nm) [24]. Robust calibrations for TSS and Antho content were reported using principal component regression (PCR), PLS, and multiple linear regression (MLR) regression models (R^2^ >= 0.90); however, lower prediction statistics were reported for flavonoids (R^2^>= 0.70) [24]. The authors also reported the use of PLS-DA to classify grapes according to the degree of maturity (classification rate > 92%) [24].

Pan and collaborators evaluated the potential of VIS-NIR spectroscopy to measure TSS and TPhenol in *Manicure Finger* and *Ugni Blanc* cultivars [25]. CIELAB, TSS, and TPhenol calibrations were developed using PLS regression. The authors reported that the developed models exhibited accuracies in the range of 77% to 94%, and concluded that the technique showed significant promise to the modern fruit and vegetable industry, particularly considering that efficient sorting and labour saving such non-destructive technologies would provide. In addition, classification models were developed to monitor time of storage (classification rates >0.77) [25].

## 3. Hyperspectral Imaging

Hernandez–Hierro and co-workers used VIS-NIR HYPER imaging to monitor total skin phenolic concentration, TSS, TA, and pH [26]. Calibration models for different grape varieties were developed, with the authors highlighting that the procedure demonstrated a great potential as a screening tool for the quality of intact whole grapes [26]. Liu and co-workers also evaluated hyperspectral images were to determine Antho content of *Cabernet Sauvignon* grown in China’s Shaanxi province [27]. The researchers collected NIR HYPER images (900–1700 nm) of grape samples and reported an R^2^ of 0.91 and RMSEP of 0.38 for the prediction of TSS content using the back-propagation neural network (BPNN) model [27].

A recent study by Gutierrez and collaborators described the use of HYPER imaging to quantify TSS and Antho content in wine grapes in the vineyard [28]. Spectra of *Tempranillo* (La Rioja, Spain) samples were collected using HYPER images under daylight with a VIS-NIR HYPER camera (400–1000 nm) [28]. Regression models for TSS yield a R^2^ of 0.91 and RMSE of 1.36 °Brix with a R^2^ of 0.92 for the prediction of external samples—RMSE of 1.27 °Brix [28]. For Antho concentration, a R^2^ of 0.72 and RMSE of 0.28 mg g^−1^ berry and 0.83 for prediction and RMSE of 0.21 mg g^−1^ berry was reported by these authors [28].

**Figure 2:**
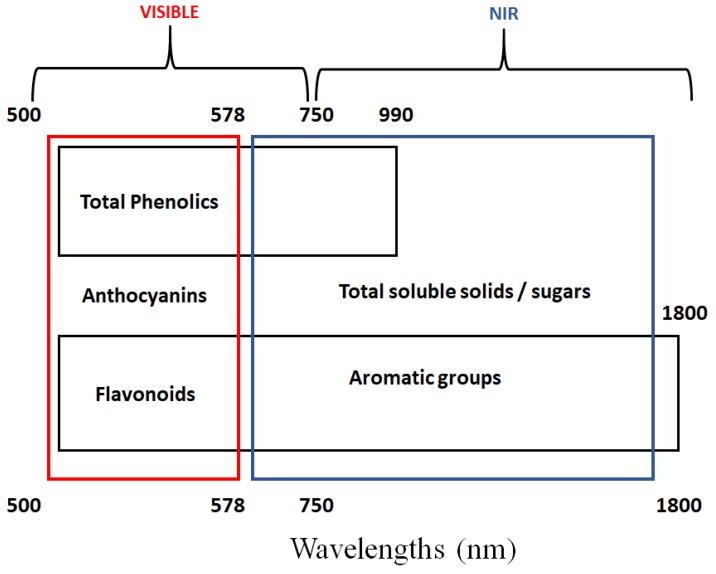
Regions in the visible and near infrared relevant to the prediction of chemical compounds in grapes. Please note that the regions are indicative and based on the information reported by other authors.

Zhang and co-authors collected NIR HYPER images during the ripening of grape samples to predict phenolic content [29]. Several algorithms were used, such as PCR, PLS, and support vector regression (SVR), to predict the TPhenol content in both grape berry and seed samples based on NIR HYPER images [29]. The authors, reported R^2^ and RMSEP values of 0.89 and 0.11 g L^−1^ for (+)-catechin equivalents (CE) for TAN in skins, 0.91 and 0.17 (g L^−1^ CE) for total iron-reactive phenolics (TIRP) in skins, 0.87 and 0.14 (g L^−1^ M3G) for Antho in skins, 0.92 and 0.24 (g L^−1^ CE) for TAN in seeds, and 0.87 and 0.51 (g L^−1^ CE) for TIRP in seeds, respectively [29]. Figure 2 summarises the main wavelengths in the visible and near infrared relevant to the prediction of chemical compounds in grapes, as reported by different authors.

## 4. Innovative applications

Volatile compounds in white *Albarino* grapes (DO Rias Baixas, Spain), analysed by gas chromatography-mass spectrometry (GC-MS) and with UV-VIS-NIR spectroscopy, was reported by Ripoll and collaborators [30]. Calibration models between the two methods where developed using PLS regression and were reported for the prediction of 2-hexenal, 1-hexanol, 2-hexanol, benzaldehyde, phenyl-ethanal, cis pyran linalool oxide and 2-phenyl-ethanol [30], with the authors highlighting that the PLS calibration models yielded a R^2^ greater than 0.85 volatile compounds measured in grape samples [30].

The content of amino acids (AA) in intact *Grenache* grape samples was evaluated by Tardaguila and co-authors using VIS and NIR spectroscopy during ripeness [31]. The best calibration models (R^2^ approx. 0.60) were reported for asparagine (SEP: 0.45 mg N L^−1^), tyrosine (SEP: 0.33 mg N L^−1^) and proline (SEP: 17.5 mg N L^−1^), for lysine (SEP: 0.44 mg N L^−1^), tyrosine (SEP: 0.26 mg N L^−1^), and proline (SEP: 15.54 mg N L^−1^) using different NIR wavelength ranges using different calibration and validation data sets, respectively [31]. In addition, the authors reported calibration models for TSS (R^2^ approx. 0.90, SEP approx. 1.60 °Brix, and RPD approx. 3.79) [31]. Rustioni and collaborators investigated the spectral properties of the epicuticular waxes of *Vitis vinifera* L. grape berries during ripening [32]. This experiment demonstrated that berry waxes have specific reflectance properties which are useful to monitor grape ripeness and maturity at harvest based on their wax content [32].

In recent years, bushfires became more frequent and intensive in viticulture regions around the world, causing contamination in grapes and wines with smoke-derived compounds (smoke taint) (e.g., USA, Australia) [33]. The lack of available practical in-field tools for the detection of smoke contamination or taint in berries prompted Fuentes and collaborators to investigate the application of a portable NIR system to monitor smoke-taint related volatile compounds in berries [33]. The researchers reported that pattern recognition allowed analysts to measure smoke taint compounds in berries with a correlation coefficient of >0.90 [33].

Table 1 showed examples of validation statistics reported by several authors on the prediction of chemical parameters in grape samples sourced from different varieties (e.g., clusters, bunches, single berries).

## 5. Summary and Future Challenges

Developments in handheld and portable instrumentation, fibre optics and hyperspectral imaging prompted the rapid evolution of the application of these technologies for the analysis of grape composition. Concomitantly, increasing computer power, easy to use software, algorithms, internet of things and data bases boosted the incorporation of these techniques into the food/wine industry.

Still some issues appeared to be recurrent in most of the reported applications. The most common/significant of these issues being the lack of information or knowledge of the standard error (SE) of the laboratory/reference method used to develop and test the calibrations models. Another is the preference of the cross validation approach to validate models rather than the use of an independent set of samples (e.g., different harvests or vintages). The introduction of modern data analysis techniques together with NIR spectroscopy can be used to monitor and quantify specific characteristics and properties in grapes and other tissues. However, correct utilisation of the process requires continuous validation and updating of the calibration models (e.g., environmental conditions, samples from different and diverse origin, appropriate spectral pre-processing) to ensure the development of a robust methodology, regrettably this critical step can be somewhat ignored or underestimated.

Savings in cost, time required to analyse the sample and the environmentally friendly nature of NIR spectroscopy makes it a very attractive technique. Developments in instrumentation, hardware and software will further position NIR as very useful tool to quantify several bioactive compounds and metabolites in fruits. With the increased availability of hand-held and portable instrumentation, the use of vibrational spectroscopy can and has been extended to in field and high-throughput methodologies for fruit metabolite analysis. Since the early 1980s, this technique has shown to be an efficient and robust alternative for the quantification and identification of metabolites and other compounds in fruits; however, some barriers exist to its widespread use. The nonexistence of academic education and formal training in NIR spectroscopy and the lack of integration of associated disciplines such as data mining and multivariate data analysis has prevented the timely implementation of these methods and technologies.

## Figures and Tables

**Table 1:** Examples of validation statistics reported by several authors on the prediction of chemical parameters in grape samples sourced from different varieties (e.g., clusters, bunches, single berries).

Parameter/Variable	N	RMSEP ^&^ or SEP ^#^	Reference
TSS (ºBrix)	114	1.011 ^&^	[34]
Anthocyanins (mg/berry)		0.618 ^&^	
Total Phenolics (au/berry)		0.749 ^&^	
Aspartic acid	89	0.36–0.46 ^#^	[31]
Glutamic acid	92	0.29–0.46 ^#^	
Asparagine	90	0.36–0.66 ^#^	
Lysine	80	0.57–0.62 ^#^	
TSS (%)	Clusters and different varieties	0.976–1.575	
Total phenolics (g/kg)		0.164–0.185	[25]
TSS	>900	1.39–1.66	[24]
Anthocyanins		16.60–18.31	
Yellow flavonoids		17.08–18.61	

Note: TSS: Total soluble solids; RMSEP: Root mean standard error of prediction; SEP: Standard error of prediction; N: Number of samples. &: RMSEP: root mean square of the standard error of prediction; #: SEP: standard error of prediction.
